# Musk (*Moschus moschiferus*) Attenuates Changes in Main Olfactory Bulb of Depressed Mice: Behavioral, Biochemical, and Histopathological Evidence

**DOI:** 10.3389/fnbeh.2021.704180

**Published:** 2021-08-27

**Authors:** Hailah M. Almohaimeed, Ashwaq H. Batawi, Zuhair M. Mohammedsaleh, Soad Al Jaouni, Samiah A. Mutlq Alsawat, Manal G. Abd El Wahab, Amany A. AbdElfattah, Nasra N. Ayuob

**Affiliations:** ^1^Department of Basic Science, Medical College, Princess Nourah Bint Abdulrahman University (PNU), Riyadh, Saudi Arabia; ^2^Department of Biological Science, Faculty of Science, King Abdulaziz University, Jeddah, Saudi Arabia; ^3^Department of Medical Laboratory Technology, Faculty of Applied Medical Sciences, University of Tabuk, Tabuk, Saudi Arabia; ^4^Department of Hematology/Pediatric Oncology, Yousef Abdullatif Jameel Chair of Prophetic Medical Applications, Faculty of Medicine, King Abdulaziz University, Jeddah, Saudi Arabia; ^5^Eye Hospital, Jeddah, Ministry of Health (MOH), Jeddah, Saudi Arabia; ^6^Department of Anatomy, Faculty of Medicine for Girls, Al-Azhar University, Cairo, Egypt; ^7^Faculty of Nurses, National Gard, King Saud University, Jeddah, Saudi Arabia; ^8^Department of Medical Histology and Cell Biology, Faculty of Medicine, Mansoura University, Mansoura, Egypt; ^9^Department of Medical Histology, Faculty of Medicine, Damietta University, Damietta, Egypt

**Keywords:** musk, olfactory bulb, depression, caspase-3, neurogenesis, GFAP, chronic stress

## Abstract

**Background:**

Musk (*Moschus moschiferus*) has been described to have a significant impact on the central nervous system, as well as anticonvulsion and antidepressant effects. This study was designed to evaluate the efficacy of musk in alleviating alterations induced in olfactory bulb of depressed mice exposed to chronic stress and identify the mechanism behind it.

**Methods:**

Fifty male albino mice were divided into five groups (*n* = 10 each): control, musk, chronic unpredictable mild stress (CUMS), fluoxetine-treated, and musk-treated groups were included in this study. Behavioral changes and serum levels of corticosterone and proinflammatory cytokines included tumor necrosis factor α, interleukin 6, and oxidant/antioxidant profile were assessed at the end of the experiment. Main olfactory bulb (MOB) has been processed for histopathological examination. Gene expression of caspase-3, glial fibrillary acidic protein, and Ki67 were assessed in the MOB using quantitative real-time polymerase chain reaction.

**Results:**

The study showed that musk inhalation significantly reduced (*p* < 0.001) corticosterone level, immobility time, inflammatory cytokines, and oxidative stress markers in CUMS-exposed mice compared to the untreated CUMS group. Musk lessened CUMS-associated neuronal alterations in the MOB and significantly reduced apoptosis and enhanced neural cell proliferation (*p* < 0.001) comparable to fluoxetine. Musk significantly enhanced the level of antioxidants in the serum and significantly reduced inflammatory cytokines. The anti-inflammatory and antioxidant activity of musk and its constituents seemed to be behind its neuroprotective effect observed in this study.

**Conclusion:**

Musk effectively ameliorated the chronic stress–induced behavioral, biochemical, and neuronal structural changes in MOB mostly through its antioxidant and anti-inflammatory effect.

## Introduction

Depression is considered a worldwide health problem as more than 264 million from different age groups, all over the world, are suffering from depression. It may promote suicide, so it is considered one of the main causes of death in people 15- to 29-year-olds (WHO, 2020).

The olfactory system is a part of the limbic system. Besides its limbic connections, olfactory system has reciprocal projections with neocortical regions and several brainstem nuclei (Fudge and Tucker, 2009). The olfactory system is known as a crucial postdevelopmental neurogenesis brain region that functions throughout life in the adult mammalian brain (Cheng et al., 2016; Cersosimo, 2018). Olfactory perceptual deficits are reported in several neurodegenerative disorders as Alzheimer disease, vascular dementia, Parkinson disease, and frontotemporal dementia. These deficits appear early and preceding the classical cognitive and motor symptoms of the disease; thus, they are considered as prodromal symptoms. These deficits are also accompanied by pathology in related brain regions, for example, the limbic system, amygdala, hippocampus, and neocortical areas (Rey et al., 2018; Yoo et al., 2018). Exposure to chronic stress has been described to impair neurogenesis in hippocampus of rodents and subsequently involved in pathogenesis of neurodegenerative disorders as major depression that has been improved by use of antidepressants (Boldrini et al., 2009; Ayuob et al., 2018). It is well evidenced that chronic unpredictable mild stress (CUMS) induces olfactory bulb (OB) dysfunction in which neurogenesis and energy metabolism disturbance are involved (Cheng et al., 2016). Moreover, olfactory bulbectomy of rodents was one of the most commonly used depression models, indicating the relationship between OB and depression (Zhou et al., 2019; Nakagawasai et al., 2020). Rodents with olfactory bulbectomy showed memory dysfunction, depressive behavior, and neurochemical changes, such as reduced hippocampal monoamines, reduced neurogenesis, and brain-derived neurotrophic factor (BDNF) levels (Odaira et al., 2019).

Exposure to psychosocial stressors has been linked to stimulation of the sympathetic nervous system, secretion of adrenaline and noradrenaline, and the release of a set of proinflammatory cytokines including interleukin 6 (IL-6) (Finnell et al., 2017). It has been reported that continuous exposure to stressors along with enhanced secretion of inflammatory cytokines results in chronic neuroinflammation that triggers depression. Adding to that, neuroinflammation disturbs the balance of the antioxidant system that augments the development of depressive state (Kim et al., 2016). Therefore, inflammatory cytokines and oxidant/antioxidant status were assessed in this study.

Complementary alternative medicine has become an attractive alternate to medications. It includes the utilization of natural materials derived from animal or plant sources in managing many health problems (Tillett and Ames, 2010). It has been reported that some essential oils might be effective in relieving emotional changes in several psychological and neurodegenerative disorders such as stress and dementia (Bikmoradi et al., 2015; Yoshiyama et al., 2015).

Musk (*Moschus moschiferus*), a substance with a penetrating odor, belongs to the well-known and valued class of the fragrance family. Natural musk has been obtained from the male musk deer (Sytniczuk et al., 2018). Musk is a part of traditional Chinese medicine as it possesses many beneficial effects such as immunity-enhancing, antibacterial, and anti-inflammatory effects (Li et al., 2018; Jie et al., 2019). Adding to that, musk has significant effects on the central nervous system (CNS) as it has been used in treating stroke, coma, neurasthenia, convulsions, and cerebral ischemia (Ayuob, 2017; Abd El Wahab et al., 2018; Lee et al., 2019; Radnaa et al., 2020). Musky compounds, for example, androstadienone, androstenol, and muscone, were described to modulate psychological state, reducing negative mood and increasing positive mood (Jacob and McClintock, 2000; Jacob et al., 2001, 2002).

The link between depression and OB has been previously described (Zhou et al., 2019; Nakagawasai et al., 2020), but the CUMS-induced impact on the main olfactory bulb (MOB) has not been clearly discussed. Therefore, the present study was designed to evaluate the impact of musk on the structural changes induced by CUMS in MOB of mice, as well as the associated biochemical and molecular changes, and explore the possible mechanisms behind these changes.

## Materials and Methods

### Chemicals

Fluoxetine (Flu) (Dar Al Dawa Pharmaceuticals Co., Ltd., Jordan) was given to the mice, at a dose of 20 mg/kg per day, through intragastric gavage and dissolved in 2 mL sodium carboxymethyl cellulose (0.03%) once per day (Li et al., 2014).

Musk (*M. moschiferus*), obtained from one of the trusted shops for musk and perfumes in Jeddah, Kingdom of Saudi Arabia (KSA), was utilized in this study. Musk compounds have been defined by gas chromatography and mass spectrometry (GC–MS) (Agilent, Columbia, SC, United States). Just before administration, musk was diluted using propylene glycol 1.0% (vol/vol) as has been previously described (Fukayama et al., 1999).

Positive control group has been exposed to amyl acetate 5% (Sigma, St. Louis, MO, United States) through inhalation. It was selected because it was safely utilized for testing olfactory detection thresholds in humans (Walker et al., 2003) and rodents (Fleming et al., 2008), and it was reported not to have an impact on anxiety (Pavesi et al., 2011).

### Animals

This experiment has been ethically approved by the Biomedical Research Ethics Committee, Faculty of Medicine, King Abdulaziz University, Jeddah, Saudi Arabia (reference no. 48-16), and followed the guidelines of animal care that has been set by King Fahd Medical Research Center (KFMRC), at King Abdulaziz University. In this study, 50 male Swiss albino mice, obtained from the animal house at KFMRC, were utilized. Their ages were ranged from 5 to 6 weeks, and their weights ranged from 30 to 40 g. They were left to acclimatize for 15 days with the laboratory conditions at 27 ± 1°C with free access to water and standard mice pellets before starting exposure to CUMS.

**FIGURE 1:**
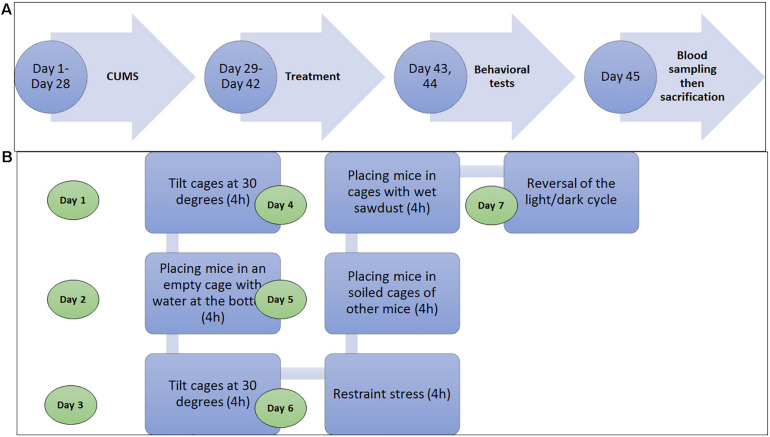
**(A)** Diagram shows that the experimental timeline shows that mice were exposed to either chronic unpredictable mild stress (CUMS) for 28 days and then treatment with amyl acetate, fluoxetine (Flu), or muck for 14. On days 43 and 44, the behavioral tests were sequentially done for all groups, and then the blood sample was obtained on the day 45, and finally, the mice were sacrificed. **(B)** Diagram shows the stressor to which the mice were exposed during the CUMS procedure in 1 week at different time points during the day. It was repeated for 4 weeks (Doron et al., 2014.

Mice were assigned, at random, into five groups (*n* = 10): control, musk, CUMS + Amyl, CUMS + Flu, and CUMS + musk groups. Each five mice were housed in a cage. The mice were exposed to CUMS protocol for 28 days according to Doron et al. (2014) followed by 2 weeks of treatment with amyl acetate, Flu, or musk. During CUMS protocol, mice were exposed to stressors at different times through the day in order to prevent adaptation to stress, for example, social stress by placing them in cages soiled by other mice, reversing the light–dark cycle, and restraining the mice. The mice were individually housed during restraint stress exposure. See the experiment timeline in Figure 1.

Administration of musk and amyl acetate has been performed through inhalation in an odor-isolated chamber (32 cm × 24 cm × 32 cm), as described (Chioca et al., 2013). Inhalation was performed once per day immediately after the CUMS procedure and lasted for 15 min, where the mice were individually placed in cages.

#### Identification of the Constituents of Musk

The components of musk have been identified by using GC–MS (Thermo Scientific, Austin, TX, United States) with a direct capillary column TR-5MS (30 m 0.25 μm 9 0.25-μm film thickness).

### Behavioral Assessment

Behavior changes were assessed at the end of the experiment in the morning (8:00–11:30 AM) through forced swimming test (FST) and elevated plus maze test (EPM) separated by 24 h (Mineur et al., 2006).

The FST is described to be sensitive to all available antidepressant drugs; therefore, it is commonly used to test the antidepressant effect of new drugs (Jeong et al., 2015). The EPM is a validated, widely used behavioral assay in rodents used to assess the antianxiety effects of pharmacological agents and to study the mechanisms underlying anxiety-related behavior (Walf and Frye, 2007). Hence, both tests were selected in this study.

Regarding FST, the total time of immobility spent by the mouse during 6 min of observation was documented in seconds. During EPM, the number of entries to closed arms was counted in 6 min, and the time spent in the open arms was estimated in seconds for each mouse using Noldus Information Technology, EthoVision XT^®^.

### Biochemical Assessment

Twenty-four hours after finishing the behavioral tests, mice were anesthetized with 4% isoflurane (SEDICO Pharmaceuticals Company, Cairo, Egypt) in 100% oxygen. Blood samples were collected from the retro-orbital venous plexus of the mice in tubes with EDTA and then centrifuged for 15 min to be kept at −80°C for biochemical assessment. Serum samples, obtained by centrifugation as previously described, were run in duplicate in order to provide enough data for statistical validation of the results. Variation coefficient, the ratio of the standard deviation to the mean, of duplicates was accepted at ≤20%.

Corticosterone level has been assessed in the serum, based on the manufacturer instructions, in order to confirm induction of depression using enzyme-linked immunosorbent assay (ELISA) kits (ALPCO Diagnostics, Orangeburg, NY, United States). Tumor necrosis factor α (TNF-α) and IL-6 (Quantikine; R&D Systems, USA Kit) were measured in the serum using ELISA as described in the manufacturer instructions.

Oxidant/antioxidant profile in the serum has been assessed through measuring malonaldehyde (MDA) spectrophotometrically at 535 nm using the thiobarbituric acid reactive substances assay kit (Biodiagnostic; Egypt) as previously described (Gamal et al., 2018). Superoxide dismutase (SOD) activity was assessed using the relevant kit (Biodiagnostic; Egypt) as has been described by Packer (2002). Glutathione peroxidase (GPX) kits (Randox Labs, Crumlin, United Kingdom) and catalase (CAT) kits (Biodiagnostic; Egypt) were also used as was described by Gamal et al. (2018).

After obtaining the blood sampling, mice were immediately euthanized by cervical dislocation, and the whole brain with the OB has been carefully dissected out, sectioned into two hemispheres, then fixed in 10% neutral-buffered formalin, and routinely processed into paraffin blocks.

Expression of Ki67, caspase-3, and glial fibrillary acidic protein (GFAP) genes in MOB was assessed using quantitative real-time polymerase chain reaction (qRT-PCR). Extraction of total RNA using Trizol from 100 mg of paraffin-processed samples of MOB was performed based on the supplier instruction (Invitrogen Life Technologies, Carlsbad, CA, United States). The methods used were previously described (Ayuob et al., 2020). The primers used are shown in Supplementary Table 1. Results are presented as the ratio of mRNA level of the gene to that of corresponding β-actin.

### Histological Findings

Paraffin-embedded brain hemispheres including the MOB were sectioned at 5-μm thickness and stained with hematoxylin-eosin (H&E) to be examined histologically using the light microscope (LM). Another set of paraffin sections was immunohistochemically stained using the peroxidase-labeled streptavidin–biotin technique. Anti-GFAP antibody (at dilution 1:1,000; Dako Cytomation, Minneapolis, MN, United States) was utilized for identification of astrocytes. Anti–caspase-3 antibody (at dilution 1:1,000; Santa Cruz Biotechnology, Santa Cruz, CA, United States) was utilized for identification of apoptosis. Anti-Ki67 antibody (at dilution 1:100; Abcam, Cambridge, United Kingdom) was used for identification of cell proliferation, whereas anti–double-cortin (DCX) (at dilution 1:100; Santa Cruz Biotechnology) has been used for neuroblast identification.

Specificity of the primary antibody was verified by preparing a negative control section with the primary antibody omitted. Slides were examined and photographed using LM connected with camera (Olympus, BX-61, Los Angeles, CA, United States). ImageJ 1.52a (National Institutes of Health, United States) was used for semiquantitative assessment of antibody immunoexpression. Area percent of GFAP immune expression, the number of caspase-3– and Ki67-positive cells, and the thickness of mitral cell layer were assessed in five fields (×400)/slide, and five slides/mouse were examined.

#### Statistical Assessment

Statistical Package for the Social Sciences (SPSS, version 22) software was used to analyze the raw data of this study. Data normality was tested using Kolmogorov–Smirnov test, and it was noticed that the data followed the normal distribution. The study variables were affected by two independent factors: stress exposure and treatment; therefore, analysis using a mixed-model two-way analysis of variance (ANOVA) based on Bonferroni *post hoc* was performed. *p* < 0.05 was considered significant.

## Results

### Compounds Detected in Musk Using GC–MS

The main compounds detected in musk, used in this study, included steroids (13.80%), essential oils (12.45%), and organic compound (41.52%), besides other compounds (Table 1). Musk compounds that were reported to have anti-inflammatory effects included steroids, α-cedrol, 5,6-dihydroaaptamine, and nerolidol, whereas those with antioxidant effects included 2, 4-di-tert-butylphenol, benzyl benzoate, isophytol, spirostan-23-ol, and others (Table 1).

**TABLE 1:** Compounds of musk identified using gas chromatography and mass spectrometer analysis.

**Compound**	RT*	**Percent of the compounds**	**Activity**
*Organic compound*		41.52	
Benzyl benzoate	26.16	6.53	– Antioxidant (Liu et al., 2012; Sriramavaratharajan and Murugan, 2018)
5-Ethyl-5-[(E)-styryl] barbituric acid	27.30	0.36	– Sedative, hypnotic, antispasmodic, anticonvulsant (Sing and Paul, 2006) – Anti-inflammatory (Sokmen et al., 2013)
5,6-Dihydroaaptamine	28.89	0.44	– Anti-inflammatory (Fristiyohadi et al., 2019)
Others		34.21	
*Steroids*		13.80	
Androstan-3-one semicarbazone	33.06	9.35	– Anti-inflammatory (Norata et al., 2010)
Spirostan-23-ol	32.16	4.46	– Anti-inflammatory, antioxidant (Lee et al., 2014; Almoulah, 2017)
*Essential oils*		12.45	
α-Cedrol	23.86	0.10	– Anti-inflammatory (Chen et al., 2020)
Isophytol	29.73	0.01	– Antioxidant, anti-inflammatory (Elsharkawy et al., 2013)
Other essential oils		12.34	
*Alcohols and phenols*		2.22	
2-(2-Hydroxypropoxy)-1-propanol	13.804	0.80	No activity reported
3,3′-Oxybis-2-butanol	14.235	0.16	– Anti-inflammatory (Kadhim et al., 2017)
2,4-Di-tert-butylphenol	21.766	0.60	– Antioxidant (Varsha et al., 2015)
2-Fluoro-6-nitrophenol	24.447	0.01	– Cytotoxic (apoptotic effect) (Al-Harthy et al., 2016)
Nerolidol	22.232	0.06	– Antioxidant, anti-inflammatory, sedative, neuroprotective (Nogueira Neto et al., 2013)
Others		0.59	
*Pyridine*		8.23	
*Terpenes*		0.09	
*Pyrans*		5.13	
Others		34.64	

*Retention time in minutes.

*The under lined words represents a category of the compound mentioned below it.*

### Behavioral Changes Induced by CUMS Exposure

In this study, statistical significance was assessed using a mixed-model two-way ANOVA to investigate the effects of two independent variables, the exposure to stress and the effect of treatment. Assessment of the behavioral changes of the mice was performed at the end of the experiment in order to confirm the occurrence of depression-like behavior after exposing mice to CUMS for 28 days. It has been found that musk inhalation did not significantly affect the behavior of the control mice during the FST or EPM test. On the other hand, CUMS significantly prolonged the immobility time (*F* = 6.34, *p* < 0.001, *n* = 10/group) during the FST. Mice of Flu-treated (*F* = 7.09, *p* = 0.03, *n* = 10/group) and musk-treated (*F* = 7.09, *p* = 0.01, *n* = 10/group) groups showed a significant reduction in immobility time compared to the CUMS group (Table 2).

**TABLE 2:** Effect of fluoxetine (Flu) and musk on the studied variables.

**Parameter (in the serum)**	**Control (*n* = 10)**	**Musk**	**CUMS + amyl (*n* = 10)**	**CUMS + flu (*n* = 10)**	**CUMS + musk (*n* = 10)**	***F***
TNF-α (pg/mL)	29.58 ± 7.83	27.27 ± 5.09 *p* = 0.96	97.17 ± 11.59 *p* < 0.001	91.23 ± 14.00 *p*# = 0.95	42.74 ± 7.69 *p*# < 0.001	*F*1 = 102.67 *F*2 = 1.51 *F*3 = 79.07
IL-6 (pg/mL)	25.97 ± 3.86	24.59 ± 4.19 *p* = 0.98	111.82 ± 11.71 *p* < 0.001	100.88 ± 17.76 *p*# = 0.22	35.39 ± 6.45 *p*# < 0.001	*F*1 = 152.62 *F*2 = 1.48 *F*3 = 134.16
MDA (nmol/mL)	1.35 ± 0.14	1.56 ± 0.60 *p* = 0.95	2.13 ± 0.64 *p* = 0.003	1.63 ± 0.44 *p*# = 0.12	1.47 ± 0.42 *p*# = 0.01	*F*1 = 5.75 *F*2 = 537.58 *F*3 = 5.78
SOD (μg/mL)	18.91 ± 2.92	24.62 ± 8.45 *p* = 0.13	12.09 ± 4.70 *p* = 0.001	13.87 ± 3.23 *p*# = 0.98	16.95 ± 3.12 *p*# = 0.03	*F*1 = 7.35 *F*2 = 752.87 *F*3 = 4.77
GPX (μg/mL)	58.60 ± 7.76	66.23 ± 10.81 *p* = 0.48	38.11 ± 6.47 *p* < 0.001	34.32 ± 5.65 *p*# = 0.97	48.19 ± 9.72 *p*# = 0.03	*F*1 = 20.57 *F*2 = 1.36 *F*3 = 8.92
CAT (μ/L)	0.41 ± 0.09	0.56 ± 0.15 *p* = 0.05	0.24 ± 0.07 *p* = 0.01	0.25 ± 0.09 *p*# = 0.98	0.43 ± 0.12 *p*# = 0.01	*F*1 = 11.02 *F*2 = 473.45 *F*3 = 11.88
Total immobility time of FST (Seconds)	308.07 ± 6.84	310.47 ± 21.60 *p* = 0.96	337.43 ± 3.18 *p* = 0.02	323.45 ± 3.47 *p*# = 0.03	316.37 ± 23.53 *p*# = 0.01	*F*1 = 6.34 *F*2 = 320.33 *F*3 = 7.09
Time spent in the open arm of EPM (Seconds)	27.80 ± 5.34	28.99 ± 6.81 *p* = 0.98	11.72 ± 3.04 *p* < 0.001	18.66 ± 4.43 *p*# = 0.01	17.86 ± 1.02 *p*# = 0.04	*F*1 = 24.49 *F*2 = 805.42 *F*3 = 8.04
No. of closed arm entries of EPM	17.33 ± 2.58	18.03 ± 2.86 *p* = 0.95	25.06 ± 1.47 *p* < 0.001	16.47 ± 3.44 *p*# < 0.001	21.40 ± 2.01 *p*# = 0.03	*F*1 = 25.44 *F*2 = 260.83 *F*3 = 30.09

*A mixed-model two-way ANOVA based on Bonferroni *post hoc* was used to analyze the data. Results are presented as mean ± SD. Significance was considered at *p* < 0.05 (*n* = 10), *df* = 39.*

*F1, effect of stress.*

*F1, effect of treatment.*

*F3, interaction between stress and treatment.*

*p, significance versus group control.*

*p#, significance versus group CUMS.*

*CUMS, chronic unpredictable mild stress; Flu, fluoxetine; IL-6, interleukin 6; TNF-α, tumor necrosis factor α; SOD, superoxide dismutase; GPX, glutathione peroxidase; CAT, catalase.*

The time spent in the open arms of EPM showed a significant decrease (*F* = 24.49, *p* < 0.001, *n* = 10/group), whereas the number of entries to the closed arms showed a significant increase (*F* = 25.44, *p* < 0.001, *n* = 10/group) in the CUMS group compared to the untreated CUMS group. Administration of Flu and musk, separately, induced a significant increase (*F* = 8.04, *p* = 0.01, *p* = 0.02, *n* = 10/group) in the time spent inside the open arms and a significant reduction (*F* = 30.09 *p* < 0.001, *n* = 10/group) in the number of entries to the closed arm compared to untreated CUMS group, respectively (Table 2).

### Biochemical Changes Induced by CUMS Exposure

#### Effect of Musk on Serum Corticosterone Level

**FIGURE 2:**
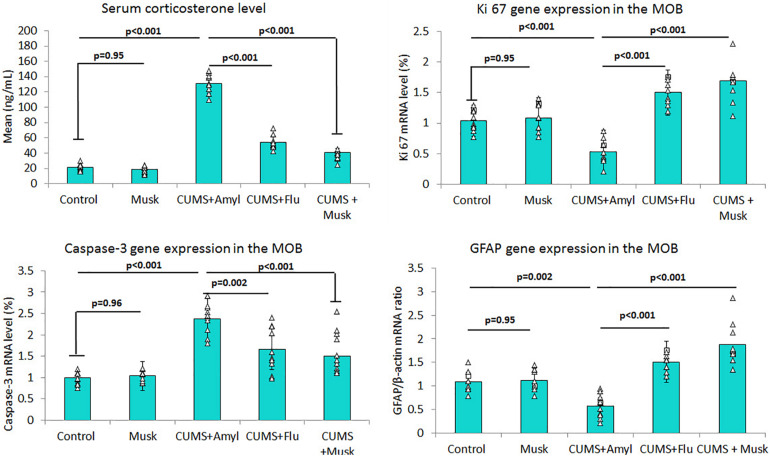
The effect of Flu and musk on serum corticosterone level, Ki67, caspase-3, and GFAP gene expression in MOB. Data are analyzed using a mixed-model two-way ANOVA based on Bonferroni *post hoc* was used to analyze the data. Results are expressed as mean ± SD (*n* = 10). *p* < 0.05 was considered significant. MOB, main olfactory bulb; CUMS, chronic unpredictable mild stress; Flu, fluoxetine.

It has been observed that musk inhalation did not significantly affect the serum corticosterone level of the control group, whereas it was significantly higher (*F* = 454.84, *p* < 0.001, *n* = 10/group) in the CUMS group than that of the control group. On the other hand, serum corticosterone was significantly lower (*F* = 399.40, *p* < 0.001, *n* = 10/group) in both Flu and musk groups compared to the untreated CUMS group (Figure 2).

#### Effect of Musk on Inflammatory Cytokines

The anti-inflammatory effect of musk has been assessed by measuring the proinflammatory cytokines levels in the serum. Although serum TNF-α and IL-6 levels did not show significant change in control mice inhaled musk, they were significantly increased (*F* = 102.67, *F* = 152.62, *p* < 0.001, *n* = 10/group) in the CUMS group compared to the control, respectively, whereas their levels showed no significant difference in the Flu-treated group. On the other hand, serum TNF-α and IL-6 levels were significantly lower (*F* = 79.07, *F* = 134.16, *p* < 0.001, *n* = 10/group) in the musk-treated group than those of the untreated CUMS group (Table 2).

#### Effect of Musk on Antioxidant Status

Serum SOD, GPX, and CAT levels showed an insignificant increase in control mice inhaled musk compared to the control group. CUMS-induced depression, in this study, was accompanied with a significant elevation (*F* = 5.75, *p* = 0.003, *n* = 10/group) in the MDA level, as well as a significant reduction in serum SOD (*F* = 7.35, *p* < 0.001, *n* = 10/group), GPX (*F* = 20.57, *p* < 0.001, *n* = 10/group), and CAT (*F* = 11.02, *p* = 0.002, *n* = 10/group) compared to the control. Whereas the musk-treated group showed significantly increased serum level of SOD (*F* = 4.77, *p* = 0.04), GPX (*F* = 8.92, *p* = 0.004), and CAT (*F* = 11.88, *p* = 0.001), the Flu-treated group did not show any significant change in their levels (Table 2).

#### Effect of Musk on Gene Expression

Levels of mRNA of caspase-3, GFAP, and Ki67 were assessed using qRT-PCR. It has been noticed that exposing mice to CUMS induced a significant up-regulation (*F* = 32.46, *p* < 0.001, *n* = 10/group) of mRNA level of caspase-3 in the MOB, while administering Flu (*F* = 13.77, *p* = 0.002) and musk (*F* = 13.77, *p* = 0.001) after exposure to CUMS induced a significant down-regulation in caspase-3 mRNA compared to the untreated group (Figure 2).

On the other hand, mRNA levels of GFAP (*F* = 43.38, *p* < 0.001) and Ki67 (*F* = 52.73, *p* < 0.001) in MOB were significantly lower in the CUMS group than those of the control group, respectively, whereas they were significantly higher in the Flu (GFAP; *F* = 61.30, *p* = 0.01, Ki67; *F* = 75.54, *p* = 0.002) and musk (*F* = 61.30, *F* = 75.54, *p* < 0.001) groups compared to the untreated CUMS group, respectively (Figure 2).

### Effect of Musk on Histopathological Changes Induced by CUMS Exposure

**FIGURE 3:**
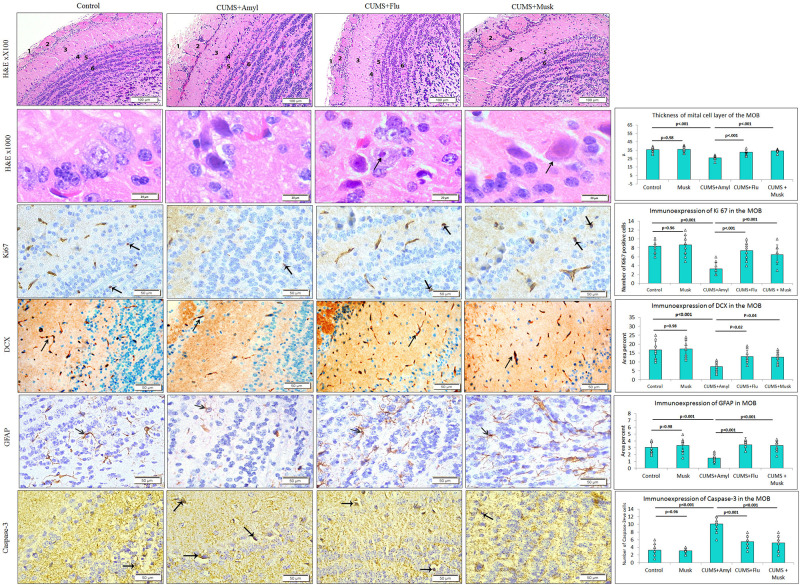
The effect of Flu and musk on the histological structure and immunoexpression of GFAP, caspase-3, Ki67, and DCX in the main olfactory bulb (MOB) of the studied groups. The MOB shows six layers; nerve fiber layer (1), glomerular layer (2), external plexiform layer (3), mitral layer (4), internal plexiform layer (5), and granular layers (6). Note the affected mitral cells in the mitral layer indicated by the arrow and surrounded by a wide pericellular space around them (arrowhead). Data are analyzed using a mixed-model two-way ANOVA based on Bonferroni *post hoc* was used to analyze the data. Results are expressed as mean ± SD (*n* = 10). *p* < 0.05 was considered significant (*df* = 39). CUMS, chronic unpredictable mild stress; Flu, fluoxetine.

Histopathological examination of the H&E-stained sections in MOB of control mice revealed regular multilayered organization of the bulb. The six distinct layers of the MOB were easily distinguished and including from inward outward: the granular cell layer, the internal plexiform layer, the mitral cell layer, the external plexiform layer, the glomerular layer, and the olfactory nerve layer, which is the outmost layer. The mitral cells, the largest cells in MOB, were triangular or multipolar nerve cells arranged in one row in the mitral cell layer. They had abundant granular basophilic cytoplasm and large pale vesicular nuclei with prominent nucleoli. There were scattered smaller granule nerve cells between the mitral cells (Figure 3). It was observed that musk inhalation did not affect the MOB of control mice (data not shown).

On the other hand, mitral cells of CUMS-exposed mice were mostly affected as they appeared distorted, shrunken with dark cytoplasm, and deeply stained pyknotic nuclei. Widening of pericellular spaces around mitral cells has been also observed (Figure 3). The thickness of the mitral cell layer was significantly reduced (*F* = 29.28, *p* < 0.001) in the CUMS group compared to that of the control mice. Flu- and musk-treated groups showed that most mitral cells restored their normal appearance except few cells that were distorted. There was a significant increase (*p* < 0.001) in the thickness of the mitral cell layer in Flu- (*F* = 24.87, *p* < 0.001) and musk-treated (*F* = 24.87, *p* < 0.001) groups compared to the CUMS group (Figure 3).

#### Immunoexpression of Caspase-3

The number of caspase-3–positive cells, observed in all layers of the MOB, was significantly higher (*F* = 39.63, *p* < 0.001) in the CUMS group compared to the control, whereas it was significantly lower (*F* = 28.07, *p* < 0.001) in Flu- or musk-treated groups compared to the untreated CUMS group with no significant difference between the two treated groups (Figure 3).

#### Immunoexpression of Ki67 and DCX

The number of Ki67- and CDX-positive cells was markedly reduced (*F* = 16.99, *F* = 14.48, *p* < 0.001) in MOB of CUMS-exposed mice compared to that of the control, respectively, whereas it was significantly higher in Flu (Ki67; *F* = 14.27, *p* < 0.001, CDX; *F* = 6.96, *p* = 0.01) and musk (Ki67; *F* = 14.27, *p* < 0.001, CDX; *F* = 6.96, *p* = 0.02) groups compared to the untreated CUMS-exposed mice, with no significant difference between the two treated groups (Figure 3).

#### Immunoexpression of GFAP

It has been observed that CUMS group showed a significant lower (*F* = 8.45, *p* < 0.001, *n* = 10/group) GFAP immunoexpression in MOB compared to the control, while it was significantly higher (*F* = 11.28, *p* < 0.001) in Flu- and musk-treated groups. No statistical significant difference existed between the two groups receiving treatment (Figure 3).

## Discussion

The olfactory sense could have unbelievable attributes if we consider its capacity to modulate human behaviors. It has determinant roles in the evolution of human habitat and in the social behavior (Sarafoleanu et al., 2009). The link between olfactory function and depression has been established. Many studies reported that decline in olfactory functions is the earliest biological marker of motor dysfunction or cognitive impairments appearing in the neurodegenerative disorders such as that occurs in Parkinson disease and Alzheimer disease (Meyer et al., 2018; Zheng et al., 2019). In this study, we hypothesized that inhalation of musk improves CUMS-induced structural changes in MOB. For confirmation of this hypothesis, mice were exposed to CUMS for a month in order to induce depression followed by exposure to musk inhalation for 2 weeks, and then CUMS-induced behavioral, biochemical, and MOB structural changes were investigated. It has been noted that exposing mice to CUMS induced a depressive state evident by prolonged immobility time during the FST and reduced time spent in the open arms of the EPM and has been verified by increased level of serum corticosterone. These findings were consistent with those previously reported (Liu et al., 2014; CB Filho et al., 2015). CUMS has been reported to increase serum corticosterone level mostly as a result of dysfunction of hypothalamic–pituitary–adrenal (HPA) axis as previously mentioned (Menard et al., 2017).

Both TNF-α and IL-6 were reported to be linked to depression pathogenesis (Taraz et al., 2015; Pedraz-Petrozzi et al., 2020); therefore, these cytokines were assessed, in this study, in order to investigate the anti-inflammatory effect of musk. We found that exposing mice to CUMS induced marked increase in inflammatory cytokines TNF-α and IL-6. This finding has been endorsed by Kim et al. (2016), who reported that the pathophysiological changes occur in depression including up-regulated proinflammatory cytokine secretion and functional resistance of glucocorticoid receptor in the hippocampus.

Stress increased oxygen free radicals in brain, which is more vulnerable to oxidative damage than other organs in addition to its limited antioxidant capacity. This resulted in damage of macromolecules such as lipid, protein, and nucleic acids, leading to neuronal dysfunction and atrophy (Freitas et al., 2014; CB Filho et al., 2015). In this study, CUMS-induced depressive-like status has been associated with disturbed antioxidant profile manifested by up-regulation of MDA and down-regulation of SOD, CAT, and GPX in the serum. Similar changes were reported by Nabavi et al. (2015) following exposure of animals to CUMS. They added that these changes were associated with oxidative stress and impairment of HPA. Liu et al. (2015) also reported that the development of depression may be due to low antioxidants content in the body.

The link between corticosterone levels, brain oxidative stress, and neuroinflammatory has been previously reported. Maternal separation model of early life stress has been reported to result in increased corticosterone levels and alterations of brain lipid peroxidation (Pinheiro et al., 2011). Brain oxidative damage has been reported to be involved in the development of CNS pathologies of neurodegenerative diseases and psychiatric disorders, in both animal models and patients (Sorce and Krause, 2009). Adding to that, reactive oxygen species (ROS) were reported to trigger alterations of brain morphology neuroinflammation and neuronal death (Gu et al., 2011).

Mitral cells of the MOB were grouped into three subtypes according to cell body shape: triangular, round, and fusiform type (Kikuta et al., 2013). Axons of mitral cells send projection to piriform cortex, the largest component of the primary olfactory cortex (Patel and Pinto, 2014). The CUMS mice, in the present work, showed structural changes in the OB in the form of mitral cell shrinkage that could be the explanation of the atrophy seen in this work in addition to other studies. Negoias et al. (2010) and Marsden (2013) reported that exposure to CUMS resulted in atrophy of limbic brain regions included hippocampus and MOB with subsequent olfactory dysfunction. CUMS exposure, in this study, led to reduction in immune and gene expression of GFAP in MOB, the selective astrocyte marker. This finding was consistent with what has been reported by Li et al. (2013), who mentioned that atrophy of microglia contributes to pathogenesis of depression.

In this study, exposing mice to CUMS induced suppression of neural cell proliferation evidenced by reduction of Ki67-positive proliferating cells as well as DCX-positive neuroblast in MOB together with the significant down-regulation of Ki67 gene expression compared to the control. Yang et al. (2011a) and Siopi et al. (2016) reported similar results in MOB using different cell proliferation markers as PSANCAM, DCX, and BrdU. Marked inhibition of neurogenesis has also been reported in the hippocampus of mice model of depression (Ayuob et al., 2018; Qi et al., 2018). This observation might be caused by elevation of plasma corticosterone in CUMS mice, which has antiproliferative effects (Ali et al., 2017; Tong et al., 2019).

Increased neuronal apoptosis has been observed in CUMS mice, in this study, manifested by numerous caspase-3–positive cells in addition to overexpression of caspase-3 gene in MOB. Previous studies have also reported similar results in rat MOB in CUMS models (Yang et al., 2011b) and in the hippocampal regions of mice subjected to CUMS (Abd El Wahab et al., 2018; Qi et al., 2018).

Fluoxetine, an approved antidepressant, has been used in this study, to relieve the depressive-like status in mice for pharmacological validation of musk. We observed that Flu attenuated the structural alteration that has been observed in MOB of CUMS mice, as well as the biochemical and the behavioral changes. Previous research reported that Flu relieved the behavioral changes and improved the structural deteriorations that occurred in the hippocampal regions of mice exposed to CUMS in accordance with our results (CB Filho et al., 2015; Ali et al., 2017; Ayuob et al., 2018). Although some studies have reported that Flu has anti-inflammatory activity (Kostadinov et al., 2015) and antioxidant properties (Caiaffo et al., 2016), in this study, administration of Flu has not been associated with a significant changes in proinflammatory cytokines or antioxidant levels. This might be attributed to the relatively short time of Flu administration.

In the present study, musk inhalation significantly improved the depressive-like behavioral changes and reduced serum corticosterone level. It also improved the neuronal affection in MOB after CUMS exposure mainly in the mitral cells in a way similar to that achieved by FLU. Thus, musk can reduce neuronal atrophy and restore astrocytes in MOB of mice. In addition, musk decreased neuronal and glial apoptosis and stimulated neural cell proliferation in mice MOB, evident by the decreased number of apoptotic cells and increased number of proliferating cells.

Similar results were reported by Abd El Wahab et al. (2018) and Ayuob et al. (2016) in the hippocampus of CUMS mice model, which is an important site for postdevelopmental neurogenesis together with MOB. Wang et al. (2015) and Lee et al. (2019) said that musk also had neuroprotective effect against focal cerebral ischemia in cases of ischemia/reperfusion injury.

These findings were also supported by those previously reported on muscone, the active ingredient of musk, which has been reported to exert antidementia (Liu et al., 2017), anti–cerebral ischemia (Surinkaew et al., 2018), and neuroprotective effects (Zhou et al., 2020). These activities and its underlying mechanisms are involved in promoting cell proliferation, ameliorating the loss of cell viability, preventing mitochondrial membrane potential collapse, lactate dehydrogenase, Ca^2+^ overload, ROS generation, and reducing the release of inflammatory factors, such as IL-1β, TNF-α, prostaglandin E2, and myeloperoxidase (Liu et al., 2017; Surinkaew et al., 2018; Zhou et al., 2020). Wei et al. (2012) and Zhang et al. (2020) reported that muscone can inhibit apoptosis *via* inhibiting expression of caspase 8 and Fas in the cortex and stimulate neural stem cells proliferation transforming them into neurons in ischemia/reperfusion rats. Muscone has also been suggested to mitigate hyperglycemia-induced Schwann cell autophagy and apoptosis partially by activating the Akt/mTOR signaling pathway (Dong et al., 2019).

This neuroprotective effect of musk might be attributed to its anti-inflammatory activity proven by the significant reduction in proinflammatory cytokines. Musk anti-inflammatory effect might be caused by its bioactive compounds detected in the present study such as steroids, α-cedrol, 5,6-dihydroaaptamine, and nerolidol. The antioxidant activity of musk, confirmed in this study, could be behind its neuroprotective effect. The compounds with antioxidant effect detected in musk, in this study, included 2,4-di-tert-butylphenol, benzyl benzoate, isophytol, spirostan-23-ol, and others. It has been reported that reduction of the antioxidants in the body may trigger the occurrence of depression (Liu et al., 2015). Therefore, restoring or enhancing the antioxidants capacity could exert a beneficial effect in preventing or treating depression.

Nerolidol, one of the compounds detected in musk in this study, has been reported to have antioxidant, anti-inflammatory, sedative, and neuroprotective effect, as it protects against hippocampal neuronal damages that occur in psychosocial disorders (Nogueira Neto et al., 2013). Nerolidol suppressed proinflammatory cytokines and inflammatory mediator release. It increased the level of SOD, CAT, and GSH and decreased the level of MDA; therefore, nerolidol treatment attenuated rotenone-induced dopaminergic neurodegeneration (Javed et al., 2016).

Among the limitations of the study was the inability to explore, in-depth, the mechanism through which musk exerts its neuroprotective effect on OB of depressed mice, although the study gave some evidence that confirmed this effect and provided some clues about the mechanism. Future study to identify the detailed mechanism is encouraged, and testing the efficacy of musk in volunteer depressed patients is also encouraged.

In conclusion, this study revealed that musk relieved behavioral depressive changes, as well as the biochemical changes observed in mice exposed to chronic mild stress. Musk also reduced apoptosis and enhanced neural cell proliferation in the MOB evidenced by underexpression of caspase-3 and enhanced expression of Ki67 gene.

## Data Availability Statement

The original contributions presented in the study are included in the article/Supplementary Material, further inquiries can be directed to the corresponding author.

## Ethics Statement

The animal study was reviewed and approved by Biomedical Research Ethics Committee, Faculty of Medicine, King Abdulaziz University, Jeddah, Saudi Arabia.

## Author Contributions

HA, AB, and ZM supervised the experiment, collect data of the investigation, and participated in results interpretation. SA, SM, MA, AA, and NA designed the experimental protocol, involved in the implementation of the overall study, performed the statistical analysis of the study, researched the data, and wrote the manuscript. All authors contributed to the revision of the manuscript and approval of the final manuscript.

## Conflict of Interest

The authors declare that the research was conducted in the absence of any commercial or financial relationships that could be construed as a potential conflict of interest.

## Publisher’s Note

All claims expressed in this article are solely those of the authors and do not necessarily represent those of their affiliated organizations, or those of the publisher, the editors and the reviewers. Any product that may be evaluated in this article, or claim that may be made by its manufacturer, is not guaranteed or endorsed by the publisher.

## Supplementary Material

Supplementary Table 1 The primers used in this study to assess gene expression through quantitative real-time polymerase chain reaction (qRT-PCR). They were obtained from Metabion International AG, Semmelweisstraße, Germany.

## Abbreviations

CUMS: chronic unpredictable mild stress
MOB: main olfactory bulb
GFAP: glial fibrillary acidic protein
BDNF: brain-derived neurotrophic factor
GC–MS: gas chromatography and mass spectrometry
FST: forced swimming test
EPM: elevated plus maze
MDA: malonaldehyde
SOD: superoxide dismutase
GPX: glutathione peroxidase
CAT: catalase.
