# Supplementary material for: Musk (Moschus moschiferus) Attenuates Changes in Main Olfactory Bulb of Depressed Mice: Behavioral, Biochemical, and Histopathological Evidence
Source: Front Behav Neurosci. 2021 Aug 27;15:704180. doi: 10.3389/fnbeh.2021.704180 (PMC8430345; doi:10.3389/fnbeh.2021.704180)
Supplement: Supplementary Table 1 — The primers used in this study to assess gene expression through quantitative real-time polymerase chain reaction (qRT-PCR). They were obtained from Metabion International AG, Semmelweisstraße, Germany. [file Table_1.pdf]

Supplementary Table (1): The primers used in this study to assess gene expression through quantitative Real-Time polymerase chain reaction (qRT-PCR). They were obtained from Metabion International AG, Semmelweisstr, Germany.

Gene	Primer sequence	
	Forward	Reverse
GFAP	“5′-CAAGCCAGACCTCACAGCG-3′”	“5′-GGTGTCCAGGCTGG-TTTCTC-3′”
Caspase-3	“5′- *TGTATGCTTACTCTACCGCACCCG* -3′”	“5′-*GCGCAAAGTGACTGGATGAACC*-3′”
Ki67	“5′-AAGAAGAGCCCACAGCACAGAGAA-3′”	“5′-AAGAAGAGCCCACAGCACAGAGAA 3′”
β-actin	“5′-TCTGGCACCACA CCTTCTA-3′”	“5′-GGCATACAGGGACAGCAC-3′”
